# Editorial: Antimicrobial Peptides - Interaction with Membrane Lipids and Proteins

**DOI:** 10.3389/fcell.2017.00004

**Published:** 2017-02-01

**Authors:** Leendert W. Hamoen, Michaela Wenzel

**Affiliations:** Bacterial Cell Biology, Swammerdam Institute for Life Sciences, University of AmsterdamAmsterdam, Netherlands

**Keywords:** antimicrobial peptides, mode of action, antibiotic resistance, membranes, artificial, membrane proteins

Antimicrobial peptides (AMPs) are a natural class of antibiotics that are present in all domains of life (Hancock and Chapple, 1999). AMPs are part of the innate immune system and possess not only antibacterial but also antiviral, antifungal, antiparasitic, and anticancer activity and often exhibit immune-modulatory properties (Zasloff, 2002). There is astonishingly little resistance to these ancient antibiotic molecules (Yeaman and Yount, 2003), despite the fact that they occur in virtually all organisms and already exist for hundreds of millions of years (Lehrer, 2013). Therefore, AMPs are considered a key weapon in fighting antibiotic-resistant pathogens (Brogden and Brogden, 2011).

One reason for this remarkable insensitivity to the buildup of resistance is that many AMPs target the cell envelope, i.e., either the cytoplasmic or outer membrane, or the peptidoglycan cell wall (Brogden, 2005). These are very complex and essential structures, which cannot easily change without substantial loss of function (Hurdle et al., 2011). Another reason is that by targeting the cytoplasmic membrane, AMPs disturb multiple processes simultaneously. E.g., the cellular response to aurein peptides showed upregulation of membrane and cell wall stress responses, fatty acid and membrane synthesis, central carbon metabolism, motility, and chemotaxis (Wenzel et al., 2015), and it has been demonstrated that inhibition of membrane function also affects metabolic pathways due to energy depletion (Spindler et al., 2011).

The vast majority of mode of action studies on AMPs has focused on their interaction with model membranes resulting in a multitude of models to explain the formation of membrane pores (Yeaman and Yount, 2003). While the “barrel-stave” (Melo et al., 2009), “toroidal pore” (van't Hof et al., 2001) or “carpet models” (Steiner et al., 1988) provide insight into interaction of amphipathic α-helical peptides with lipid bilayers, the “molecular electroporation” (Miteva et al., 1999), “sinking raft” (Pokorny et al., 2002), “interfacial activity” (Wimley, 2010), and “lipocentric pore formation” (Fuertes et al., 2011) models are more applicable for small, unstructured, or compact peptides that are unable to span the lipid bilayer to form a pore directly. However, there is increasing evidence that many peptides are not forming pores in bacteria at all and that the membrane disruption observed *in vitro* is only part of the picture (Scheinpflug et al., 2015). The hexapeptide RWRWRW-NH_2_ was shown to inhibit cell wall synthesis and respiration by displacing involved proteins from the membrane (Wenzel et al., 2014), and the last resort lipopeptide antibiotic daptomycin was found to insert into fluid membrane microdomains and delocalize phospholipid and cell wall synthesis enzymes (Müller et al., 2016). Thus, in order to thoroughly understand the mechanisms of action of AMPs, it is necessary to view biological membranes as a whole, including membrane organization and protein localization. The articles in this collection cover studies on AMP interaction with membrane components both *in vitro* and *in vivo* as well as boosting AMP activity and evaluating their cytotoxicity.

One major challenge in the development of AMPs as novel drugs is enhancing their activity against Gram-negative bacteria. A nice example is the study by Bluhm et al., who were able to optimize apidaecins, insect-derived peptides that interfere with ribosome assembly, toward higher activity against *Pseudomonas aeruginosa*, which has an intrinsically high antibiotic resistance and is on its way to pan-resistance. In fact, it is an important limitation of many antibiotics, not only AMPs, that they are not very effective against Gram-negative bacteria, a consequence of their outer membrane barrier. Zhou et al. addressed this issue by fusing the amino acid sequences of peptides that are known to permeate the outer membrane to the C-terminus of the potent lantibiotic nisin, generating molecules with two-fold enhanced activity against Gram-negatives. Lantibiotics are an extremely interesting class of AMPs, since many of them specifically bind cell wall precursors to exert their membrane activity, resulting in remarkable selectivity for bacterial over mammalian cells (Schneider and Sahl, 2010). While many new peptides are screened for cytotoxicity, it has now been demonstrated that this initial screening needs to be improved. A case in point is the hexapeptide RWRWRW-NH_2_, which was not toxic or hemolytic in earlier studies (Albada et al., 2012), but failed the acute toxicity test in mice. Although it only leads to minor release of hemoglobin from erythrocytes, which was interpreted as mild hemolytic potential, damaged erythrocyte membranes can easily be observed under the microscope (Wenzel et al.).

The mechanism of AMPs has been investigated on the molecular level by Ciesielski et al., who provided new insight into how amphotericin B and natamycin recognize membrane sterols using ^13^C MAS NMR (Ciesielski et al.), and by Grage et al., who showed that membrane thinning, which has been proposed as part of the mode of action of AMPs (Grage et al., 2010), is a valid concept for some peptides but depends on both the individual peptide and the lipid bilayer and cannot be generalized (Grage et al.). For cellular mode of action studies, te Winkel et al. provided detailed guidance on how to analyze the capacity of membrane-active compounds to dissipate the membrane potential in bacteria using voltage-sensitive dyes. The broad range of antibacterial mechanisms of AMPs and the stress response of bacteria toward these molecules has been extensively reviewed by Omardien et al. Finally, the growing evidence that AMPs are not just unselectively disrupting membrane bilayers but actually targeting specialized membrane foci is discussed by Rashid et al.

In conclusion, AMPs are a structurally and mechanistically diverse group of promising antibiotic agents with great clinical potential. More and more evidence is emerging that their mechanisms involve a much wider variety of interactions with membrane components than previously assumed. This research topic has brought together different viewpoints of AMP research from molecules to systems and we hope that this collection will promote future collaborations across fields to investigate these promising compounds.

## Author contributions

All authors listed, have made substantial, direct and intellectual contribution to the work, and approved it for publication.

## Funding

LH was financially supported by the Netherlands Organization for Scientific Research (NWO, http://www.nwo.nl/en, STW-Vici 12128).

### Conflict of interest statement

The authors declare that the research was conducted in the absence of any commercial or financial relationships that could be construed as a potential conflict of interest.

## References

[B1] Albada H. B. Chiriac A. I. Wenzel M. Penkova M. Bandow J. E. Sahl H. G.. (2012). Modulating the activity of short arginine-tryptophan containing antibacterial peptides with N-terminal metallocenoyl groups. Beilstein J. Org. Chem. 8, 1753–1764. 10.3762/bjoc.8.200 23209509 PMC3511009

[B2] Brogden K. A. (2005). Antimicrobial peptides: pore formers or metabolic inhibitors in bacteria? Nat. Rev. Microbiol. 3, 238–250. 10.1038/nrmicro1098 15703760

[B3] Brogden N. K. Brogden K. A. (2011). Will new generations of modified antimicrobial peptides improve their potential as pharmaceuticals? Int. J. Antimicrob. Agents 38, 217–225. 10.1016/j.ijantimicag.2011.05.004 21733662 PMC3159164

[B4] Fuertes G. Giménez D. Esteban-Martín S. Sánchez-Mu-oz O. L. Salgado J. (2011). A lipocentric view of peptide-induced pores. Eur. Biophys. J. 40, 399–415. 10.1007/s00249-011-0693-4 21442255 PMC3070086

[B5] Grage S. L. Afonin S. Ulrich A. S. (2010). Dynamic transitions of membrane-active peptides. Methods Mol. Biol. 618, 183–207. 10.1007/978-1-60761-594-1_13 20094866

[B6] Hancock R. E. Chapple D. S. (1999). Peptide antibiotics. Antimicrob. Agents Chemother. 43, 1317–1323. 10348745 10.1128/aac.43.6.1317 PMC89271

[B7] Hurdle J. G. O'Neill A. J. Chopra I. Lee R. E. (2011). Targeting bacterial membrane function: an underexploited mechanism for treating persistent infections. Nat. Rev. Microbiol. 9, 62–75. 10.1038/nrmicro2474 21164535 PMC3496266

[B8] Lehrer R. (2013). Evolution of antimicrobial peptides: A view from the cystine chapel, in Antimicrobial Peptides and Immunity, eds Hiemstra P. Zaat S. (Basel: Springer), 1–25.

[B9] Melo M. N. Ferre R. Castanho M. A. R. B. (2009). Antimicrobial peptides: linking partition, activity and high membrane-bound concentrations. Nat. Rev. Microbiol. 7, 245–250. 10.1038/nrmicro2095 19219054

[B10] Miteva M. Andersson M. Karshikoff A. Otting G. (1999). Molecular electroporation: a unifying concept for the description of membrane pore formation by antibacterial peptides, exemplified with NK-lysin. FEBS Lett. 462, 155–158. 10.1016/S0014-5793(99)01520-3 10580110

[B11] Müller A. Wenzel M. Strahl H. Grein F. Saaki T. Kohl B.. (2016). Daptomycin inhibits bacterial cell envelope synthesis by interfering with fluid membrane microdomains. Proc. Natl. Acad. Sci. U.S.A. 113, E7077–E7086. 10.1073/pnas.1611173113 27791134 PMC5111643

[B12] Pokorny A. Birkbeck T. H. Almeida P. F. F. (2002). Mechanism and kinetics of delta-lysin interaction with phospholipid vesicles. Biochemistry 41, 11044–11056. 10.1021/bi020244r 12206677

[B13] Scheinpflug K. Krylova O. Nikolenko H. Thurm C. Dathe M. (2015). Evidence for a novel mechanism of antimicrobial action of a cyclic R-,W-rich hexapeptide. PLoS ONE 10:e0125056. 10.1371/journal.pone.0125056 25875357 PMC4398456

[B14] Schneider T. Sahl H. G. (2010). An oldie but a goodie - cell wall biosynthesis as antibiotic target pathway. Int. J. Med. Microbiol. 300, 161–169. 10.1016/j.ijmm.2009.10.005 20005776

[B15] Spindler E. C. Hale J. D. Giddings T. H. Hancock R. E. Gill R. T. (2011). Deciphering the mode of action of the synthetic antimicrobial peptide Bac8c. Antimicrob. Agents Chemother. 55, 1706–1716. 10.1128/AAC.01053-10 21282431 PMC3067151

[B16] Steiner H. Andreu D. Merrifield R. B. (1988). Binding and action of cecropin and cecropin analogues: antibacterial peptides from insects. Biochim. Biophys. Acta 939, 260–266. 10.1016/0005-2736(88)90069-7 3128324

[B17] van't Hof W. Veerman E. C. Helmerhorst E. J. Amerongen A. V. (2001). Antimicrobial peptides: properties and applicability. Biol. Chem. 382, 597–619. 10.1515/BC.2001.072 11405223

[B18] Wenzel M. Chiriac A. I. Otto A. Zweytick D. May C. Schumacher C.. (2014). Small cationic antimicrobial peptides delocalize peripheral membrane proteins. Proc. Natl. Acad. Sci. U.S.A. 111, E1409–E1418. 10.1073/pnas.1319900111 24706874 PMC3986158

[B19] Wenzel M. Senges C. H. Zhang J. Suleman S. Nguyen M. Kumar P.. (2015). Antimicrobial peptides from the aurein family form ion-selective pores in *Bacillus subtilis*. ChemBioChem 16, 1101–1108. 10.1002/cbic.201500020 25821129

[B20] Wimley W. C. (2010). Describing the mechanism of antimicrobial peptide action with the interfacial activity model. ACS Chem. Biol. 5, 905–917. 10.1021/cb1001558 20698568 PMC2955829

[B21] Yeaman M. R. Yount N. Y. (2003). Mechanisms of antimicrobial peptide action and resistance. Pharmacol. Rev. 55, 27–55. 10.1124/pr.55.1.2 12615953

[B22] Zasloff M. (2002). Antimicrobial peptides of multicellular organisms. Nature 415, 389–395. 10.1038/415389a 11807545

